# Therapeutic regimen of Crohn’s disease: effect of Infliximab combined with mesalazine on intestinal flora and inflammatory indexes in patients

**DOI:** 10.1186/s12876-025-04236-9

**Published:** 2025-09-29

**Authors:** Xiaoxia Xiang, Zhenghua Hu, Siyuan Dong, Bo Qi, Angyan Li, Liping Ji

**Affiliations:** 1 Department of Gastroenterology, Haiyan People’s Hospital, Jiaxing City, China; 2 Department of Gastroenterology, Sir Run Run Shaw Hospital, Zhejiang University School of Medicine, Hangzhou City, 310000 China

**Keywords:** Crohn's disease, Infliximab, Mesalazine, Intestinal flora, Inflammation

## Abstract

**Objective:**

To study the clinical efficacy of infliximab (IFX) in combination with mesalazine in patients with Crohn’s disease (CD).

**Methods:**

This retrospective analysis study included 197 patients with CD who were treated in Haiyan People's Hospital and Sir Run Run Shaw Hospital, School of Medicine, Zhejiang University our hospital between March 2023 and March 2024, and were divided into two groups according to the different treatments recorded in the electronic medical record system: 82 cases in the control group (mesalazine) and 115 cases in the observation group (IFX combined with mesalazine treatment). The intestinal flora (bifidobacteria, Lactobacillus, Enterococcus, Enterobacteriaceae), serum indicators (tumor necrosis factor-α (TNF-α), C-reactive protein (CRP), erythrocyte sedimentation rate (ESR), hemoglobin concentration (HGB), platelets (PLT), Th1, Th2, Th1/Th2), major symptoms and stool status (disappearance of abdominal pain, disappearance of diarrhea, stool frequency and stool normalization), clinical efficacy and complication situation were evaluated.

**Results:**

The number of bifidobacteria and lactobacilli in the observation group was higher than that in the control group, and the difference was statistically significant (*P* < 0.05); TNF-α, CRP, ESR, PLT, Th1 and Th1/Th2 were lower than those in the control group, but HGB and Th2 were higher than those in the control group, and the difference was statistically significant (*P* < 0.05); After treatment, the rate of abdominal pain disappearance, diarrhea disappearance and normal stool rate were higher than that of the control group, and the number of stools was lower than that of the control group, with statistically significant differences (*P* < 0.05); the total clinical effective rate of the observation group (92.17% vs. 80.49%) was higher than that of the control group, with statistically significant difference (*P* < 0.05); the overall incidence rate of adverse reactions of the observation group (8.70% vs. 7.32%) was higher than that of the control group, but the difference was not statistically significant (*P* > 0.05).

**Conclusion:**

IFX combined with mesalazine demonstrates superior clinical efficacy compared to mesalazine monotherapy in patients with Crohn’s disease. This combination effectively improves intestinal microbiota balance, reduces inflammation, modulates immune responses, and alleviates clinical symptoms without significantly increasing adverse events.

**Supplementary Information:**

The online version contains supplementary material available at 10.1186/s12876-025-04236-9.

## Introduction

Crohn’s disease (CD), as a chronic, recurrent inflammatory bowel disease, has shown a significant increase in its global incidence over the past two decades, especially in industrialized countries, with an annual growth rate as high as 3–4% [1–3]. Honap S et al. [4] stated that the disease is characterized by transmural inflammation, segmental intestinal injuries, and a high prevalence of complications (e.g., intestinal fistulas, strictures, and abscesses), with the clinical symptoms of abdominal pain, diarrhea, loss of body mass, etc. It can involve any part of the entire digestive tract from the mouth to the anus, causing damage to the entire intestinal wall, and the site of onset is often located in the terminal ileum and the right half of the colon, combined with extraintestinal pathologies such as peripheral arthritis, erythema nodosum, and perianal lesions, and in some patients, perianal lesions and fistulas are the first symptom, which severely affects the quality of life of patients and the social burden of healthcare. Several studies have indicated that although the interaction of genetic susceptibility, immune dysregulation, environmental factors and intestinal flora disorders is recognized as the core mechanism for the pathogenesis of CD, the exact pathophysiological network has not yet been fully elucidated, resulting in clinical treatments that still face significant challenges such as insufficient remission rates, high recurrence rates and drug resistance [5, 6].

Currently, CD treatment strategies have shifted from traditional symptom control to precision-targeted therapies based on disease stratification. Infliximab (IFX), an anti-Tumor Necrosis Factor (TNF)-α IgG1-κ chimeric monoclonal antibody, as a representative of the biologics, has significantly altered the treatment of moderate-to-severe CD through the neutralization of the pro-inflammatory effects of TNF-α, induction of mucosal healing, and modulation of immune cell apoptosis [7, 8]. Louis E et al. [9] stated that IFX, the first biologic agent for the treatment of CD, was initially approved for the treatment of active CD in children in the United States in 2006; however, clinical practice has shown that the overall response rate to single IFX therapy is only 60%−70%, and secondary loss of response occurs in about 30%−40% of the patients within 1 year of treatment, and this limitation is partly attributed to the complex immunogenicity of the drug, differences in metabolic transformation mediated by intestinal flora, and compensatory activation of inflammatory signaling pathways. Therefore, exploring synergistic treatment regimens of IFX with other drugs becomes a key breakthrough to improve clinical remission rates and delay the onset of drug resistance. Jhakri K and other [10] scholars concluded that mesalazine belongs to the 5-aminosalicylic acid (5-ASA) class of drugs, and as a first-line therapeutic agent for mild to moderate CD, its local anti-inflammatory effect relies on the mechanisms of inhibition of cyclooxygenase (COX), blockade of prostaglandin synthesis, and scavenging of free radicals, and it can inhibit the inflammatory response of intestinal mucosa via the gastric acidic environment and into the intestinal microenvironment. Although several early studies have questioned the efficacy of 5-ASA in moderate-to-severe CD, recent basic research has revealed that it can exert synergistic anti-inflammatory effects by regulating the expression of intestinal epithelial tight junction proteins, inhibiting the nuclear factor-κB (NF-κB) pathway, and regulating the structure of intestinal flora [11–13]. This suggests that there may be an under-explored synergistic mechanism for the combination of the two drugs, but the dynamic effects of this combination therapy on intestinal microecological remodeling and its causal relationship with inflammation alleviation have rarely been reported clinically. As a “key regulator” of CD pathology, dysregulation of the intestinal flora can drive chronic inflammation by disrupting the integrity of the mucosal barrier, activating aberrant immune responses, and metabolite imbalance [14]. Pro-inflammatory genera were elevated in abundance, while anti-inflammatory flora such as short-chain fatty acid-producing Rousella and Clostridium pumilus were significantly reduced. Mesalazine was able to inhibit the biofilm formation of pathogenic Escherichia coli and promote the proliferation of butyric acid-producing bacteria, and at the level of inflammation modulation, IFX may potentially inhibit the over-activation of the Th1/Th17 pathway by inducing the differentiation of regulatory T-cells (Tregs), while mesalazine may reduce intestinal inflammation by inhibiting the IL-6/STAT3 signaling to enhance mucosal repair. However, there is no clinical study systematically revealing the modulation pattern of IFX combined with mesalazine on the diversity of intestinal flora and functional gene profiles in CD patients, not to mention the lack of correlation analysis with serum inflammatory markers. Recent systematic reviews have highlighted the evolving landscape of biological therapies in inflammatory bowel disease, supporting the rationale for combination therapy approaches [32, 33].

Based on this, the present study is based on the scientific question of the synergistic mechanism of biological agents and traditional drugs in CD treatment, and analyzes the potential effects of IFX combined with mesalazine on patients, aiming to provide a theoretical basis for optimizing the precise combination therapy strategy for CD.

## Materials and methods

### Statement of ethics

This study was approved by our Institutional Review Board and Ethics Committee (IRB No. 52336). Given that this study was retrospective and only de-identified patient data were used, informed consent was not required as there was no risk or adverse effect on patient care. This waiver is in line with regulatory and ethical guidelines related to retrospective studies. Haiyan People’s Hospital and Sir Run Run Shaw Hospital waived the need for informed consent.

### Study design

This retrospective analytic study included 197 patients with CD who were treated between March 2023 and March 2024 at Haiyan People's Hospital and Sir Run Run Shaw Hospital, School of Medicine, Zhejiang University.our hospital. They were divided into two groups according to the different treatments recorded in the electronic medical record system: 82 cases in the control group (mesalazine) and 115 cases in the observation group (IFX combined with mesalazine treatment).

### Inclusion criteria

Inclusion criteria: (1) age > 18 years old; (2) clinical manifestations and endoscopic and pathological findings meet the criteria in the Evidence-Based Clinical Practice Guidelines for Inflammatory Bowel Disease [16] and confirmed the diagnosis; (3) those who fail to achieve complete remission or are intolerant to drugs after treatment with traditional medications (including aminosalicylic acid agents, glucocorticosteroids, etc.) or enteral nutrition; (4) clinical and follow-up data are complete; and (5) those who do not have concomitant neurological disease or cognitive dysfunction.

Exclusion criteria: (1) active infection; (2) intestinal CD combined with fibrous stenotic obstruction without evidence of inflammatory response; (3) other complications, drug allergy, contraindications to the drugs used in this experiment, and recent use of hormonal drugs or immunosuppressive drugs; (4) pregnant and lactating women; (5) combined with serious digestive and circulatory system diseases.

### Treatment method

Both groups were given conventional treatment, such as nutritional support, correction of electrolyte disorders, regulation of intestinal flora, intestinal mucosal protection. On this basis, the control group was orally administered Mesalazine Sustained-release Tablets (manufacturer: Huilin International Pharmaceutical (Switzerland) Co., LTD) batch no.: State Pharmaceutical License No. H20100609, specification: 0.5g) ，1 g/times, 3 times/d.On this basis, the control group was orally administered mesalazine enteric-coated tablets (manufacturer: Sunflower Pharmaceutical Group Jiamusi Luling Pharmaceutical Co., Ltd, batch no.: State Pharmaceutical License No. H19980148, specification: 0.25 g) 0.5 g/times, 3 times/d.

The observation group was treated with IFX in combination with IFX for injection (manufacturer: Taizhou MaiBoTaiKe Pharmaceutical Co., Ltd, batch number: State Drug Permit S20210025, specification: 100 mg) on the basis of the control group in accordance with the “Recommended Protocol for the Treatment of Crohn’s Disease by Infliximab” [17], the first dose was injected at 5 mg/kg, and the second and the third doses were administered in the second and sixth weeks, respectively, and the patients were observed for their response to the drug, if the condition is not well controlled, increase the dose to 10 mg/kg injection, each drug was dissolved with 10 mL of sterile water for injection, and then diluted to 250 mL with 0.9% sodium chloride injection, the infusion time should not be less than 2 h, once every 8 weeks, a total of 7 times.

All patients were treated for a period of 40 weeks, and the relevant indexes were collected on the day of admission and on the 2nd day after the end of the 40-week treatment.

### General information

General demographic data of the patients including age, sex (male/female), body mass index, site of lesion (terminal ileum/colon/ileocecum), duration of the disease, family history (yes/no) were collected through medical record system.

### Number of intestinal flora

Fresh fecal samples were collected from the two groups of patients before and after treatment, well preserved, and then sent to the examination room, appropriate amount of samples were implanted into the culture medium, and proliferated in a constant temperature of 37℃ for 24 h. Then the colonies in the medium were observed, and the numbers of bifidobacteria, lactobacillus, enterococci, enterobacteria, etc. were mainly determined. Additionally, fecal samples were stored at −80℃ and sent to a third-party laboratory for 16 S rRNA gene sequencing analysis. The present manuscript reports the culture-based results for the four major bacterial groups, while sequencing data and α/β diversity analyses are provided in the supplementary materials (Table S1 Figure S1).

### Serum indicators

Before and after treatment, collect 5 ml of morning fasting venous blood from patients, use centrifuge (model: LL900, manufacturer: Luoyang Hongshi Machinery Equipment Co., Ltd.), 3000r/min, centrifuge for 10 min, separate the patient’s serum, and use enzyme-linked immunoassay to determine TNF-α, C-reactive protein (CRP), erythrocyte sedimentation rate (ESR), hemoglobin concentration (HGB), platelet (PLT), Th1, Th2, and Th1/Th2 levels were determined from the same lot of kit, which was in the qualification period, and the specific values in the samples were determined.

### Main symptoms and stool condition

After treatment, clinical observation was made to assess the disappearance of abdominal pain, disappearance of diarrhea, number of stools and normal stool condition (no dilute stools, mucus stools, mucus-blood stools, bloody stools, watery stools) of the patients, and comparisons were made.

### Clinical efficacy

The efficacy of the two groups of patients was evaluated at the end of treatment, based on the changes in abdominal pain, diarrhea and other symptoms and the Crohn’s Disease Activity Index (CDAI) [18]. The CDAI is calculated based on eight clinical variables: (1) number of liquid or soft stools per day × 2; (2) abdominal pain (0 = none, 1 = mild, 2 = moderate, 3 = severe) × 5; (3) general well-being (0 = well, 1 = slightly below par, 2 = poor, 3 = very poor, 4 = terrible) × 7; (4) presence of complications × 20; (5) use of diphenoxylate or opiates for diarrhea × 30; (6) presence of abdominal mass (0 = none, 2 = questionable, 5 = definite) × 10; (7) hematocrit deficit in percentage points × 6; (8) percentage deviation from standard body weight × 1. Clinical outcomes were defined as follows: Clinical remission: CDAI < 150; Clinical response: decrease in CDAI score ≥ 100 points; No response/relapse: failure to meet the above criteria or increase in CDAI score > 70 points. Overall effective rate = (clinical remission + clinical response)/number of cases × 100%.

### Occurrence of adverse reactions

Observe and record the occurrence of adverse reactions such as rash, dizziness, nausea, vomiting and so on during the treatment of the two groups of patients.

### Statistical processing

This paper uses SPSS 25.0 statistical software to calculate the data, the count data is expressed by [n(%)], and the χ² test is adopted; the measurement data is tested by the Shapiro-Wilk method to be in line with the normal distribution, and it is expressed by (mean ± SD), and the comparison between the two groups is made by the t-test of independent samples, and the comparison within the group is made by the t of paired samples. For multiple comparisons, Bonferroni correction was applied with a significance threshold set at 0.05/13 ≈ 0.004. All significant findings reported in this manuscript met this corrected threshold. Based on the primary endpoint (CDAI decrease ≥ 70), a sample size of 197 patients provided > 90% power to detect an effect size of 0.5 at α = 0.05.

### Statistical control strategies

To minimize confounding bias inherent in the retrospective design, we employed multivariate logistic/linear regression models adjusting for key covariates including age, disease duration, baseline CDAI scores, and previous medication history. Additionally, inverse probability of treatment weighting (IPTW) based on propensity scores was performed to verify the robustness of main outcomes.

## Results

### Comparison of baseline data of CD patients in two groups

Comparison of baseline data such as age, gender, body mass index, lesion site, disease duration, family history and other data of patients in the observation group and the control group, the differences are not statistically significant (*P* > 0.05), suggesting that the groups are comparable, see Table 1.

**Table 1:** Comparison of baseline data of two groups of patients with CD

Index	Observation group (*n* = 115)	Control group (*n* = 82)	χ²/t value	*P* value
Age (years)	31.93 ± 5.96	31.56 ± 5.57	0.441	0.660
Sex (n, %)			0.054	0.816
Male	72 (62.61)	50 (60.98)		
Female	43 (37.39)	32 (39.02)		
Body mass index (kg/m²)	23.75 ± 1.48	23.39 ± 1.56	1.645	0.101
Lesion site (n, %)			0.081	0.960
Terminal ileum	45 (39.13)	31 (37.80)		
Colon	10 (8.70)	8 (9.76)		
Ileocolon	60 (52.17)	43 (52.44)		
Course of disease (years)	7.20 ± 2.98	7.12 ± 2.55	0.197	0.844
Family history (n, %)			0.065	0.799
Have	10 (8.70)	8 (9.76)		
No	105 (91.30)	74 (90.24)		

### Comparison of the number of intestinal flora between the two groups of CD patients

Intestinal flora is closely associated with the body’s immune system and inflammation, reflecting the body’s intestinal function, immune response, etc., which has an important impact on the efficacy of CD patients. Before treatment, the comparison of the number of bifidobacteria, lactobacillus, enterococci and enterobacteria in the observation group and the control group, the difference is not statistically significant (*P* > 0.05), see Table 2.

**Table 2:** Comparison of intestinal flora quantity between two groups of CD patients before treatment (mean ± SD, log₁₀ CFU/g)

Index	Observation group (*n* = 115)	Control group (*n* = 82)	t value	*P* value
Bifidobacterium	5.46 ± 0.76	5.53 ± 0.79	0.627	0.531
Lactobacillus	5.46 ± 0.83	5.33 ± 0.88	1.057	0.292
Enterococcus	8.06 ± 1.13	8.12 ± 1.25	0.351	0.726
Enterobacter	8.31 ± 1.26	8.24 ± 1.31	0.378	0.706

**Fig. 1:**
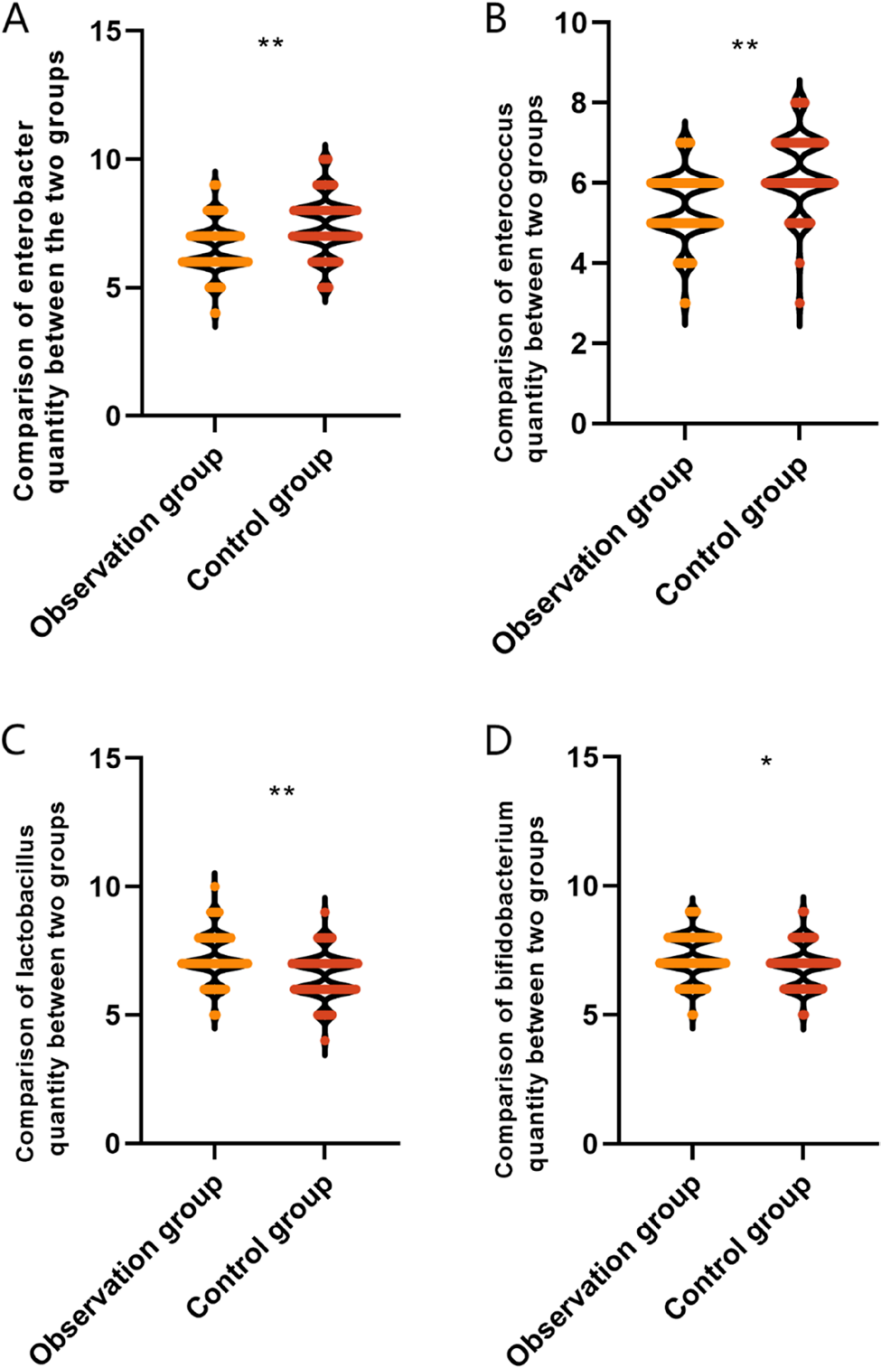
Comparison of intestinal flora in two groups of CD patients after treatment Note; Data are presented as mean ± SD (log₁₀ CFU/g). *P < 0.05, **P < 0.001

After treatment, the number of bifidobacteria (7.21 ± 0.86 vs. 6.88 ± 0.81 log₁₀ CFU/g) and lactobacilli (7.19 ± 0.92 vs. 6.51 ± 0.90 log₁₀ CFU/g) in the observation group was higher than that in the control group, but the number of enterococci (5.42 ± 0.83 vs. 6.26 ± 0.94 log₁₀ CFU/g), enterobacteria (6.51 ± 0.96 vs. 7.39 ± 1.12 log₁₀ CFU/g) were lower than those in the control group, and the difference was statistically significant (*P* < 0.001), demonstrating that the combination of IFX and mesalazine led to a more favorable modulation of gut flora, as shown in Fig. 1. After IPTW adjustment, the direction and significance of main outcomes remained unchanged (see Supplementary Table S2.

### Comparison of serum indexes between the two groups of CD patients

TNF-α, CRP, ESR are important indicators for the assessment of the body’s inflammatory response, HGB, PLT reflect the coagulation status of CD patients, and Th1, Th2, Th1/Th2 are important cell subpopulations in the adaptive immune response, reflecting the body’s immune response. Before treatment, TNF-α, CRP, ESR, HGB, PLT, Th1, Th2, Th1/Th2 were compared between patients in the observation group and the control group, and the differences were not statistically significant (*P* > 0.05), see Table 3.

**Table 3:** Comparison of serum indexes before treatment between two groups of CD patients (mean ± SD)

Index	Observation group (*n* = 115)	Control group (*n* = 82)	t value	*P* value
TNF-α (ng/L)	23.16 ± 4.16	22.81 ± 4.39	0.569	0.570
CRP (mg/L)	38.21 ± 5.72	37.89 ± 5.98	0.380	0.705
ESR (mm/h)	41.33 ± 6.78	41.12 ± 6.03	0.224	0.823
HGB (g/L)	96.14 ± 12.52	96.28 ± 12.17	0.078	0.938
PLT (×10⁹/L)	428.10 ± 69.85	426.49 ± 72.85	0.157	0.876
Th1 (%)	42.63 ± 3.58	42.71 ± 3.46	0.162	0.871
Th2 (%)	26.95 ± 2.18	26.88 ± 2.27	0.218	0.827
Th1/Th2	1.60 ± 0.58	1.54 ± 0.53	0.742	0.459

Note: *TNF*-α is tumor necrosis factor-α, *CRP* is C-reactive protein, *ESR* is erythrocyte sedimentation rate, *HGB* is hemoglobin concentration, and *PLT* is platelets

**Fig. 2:**
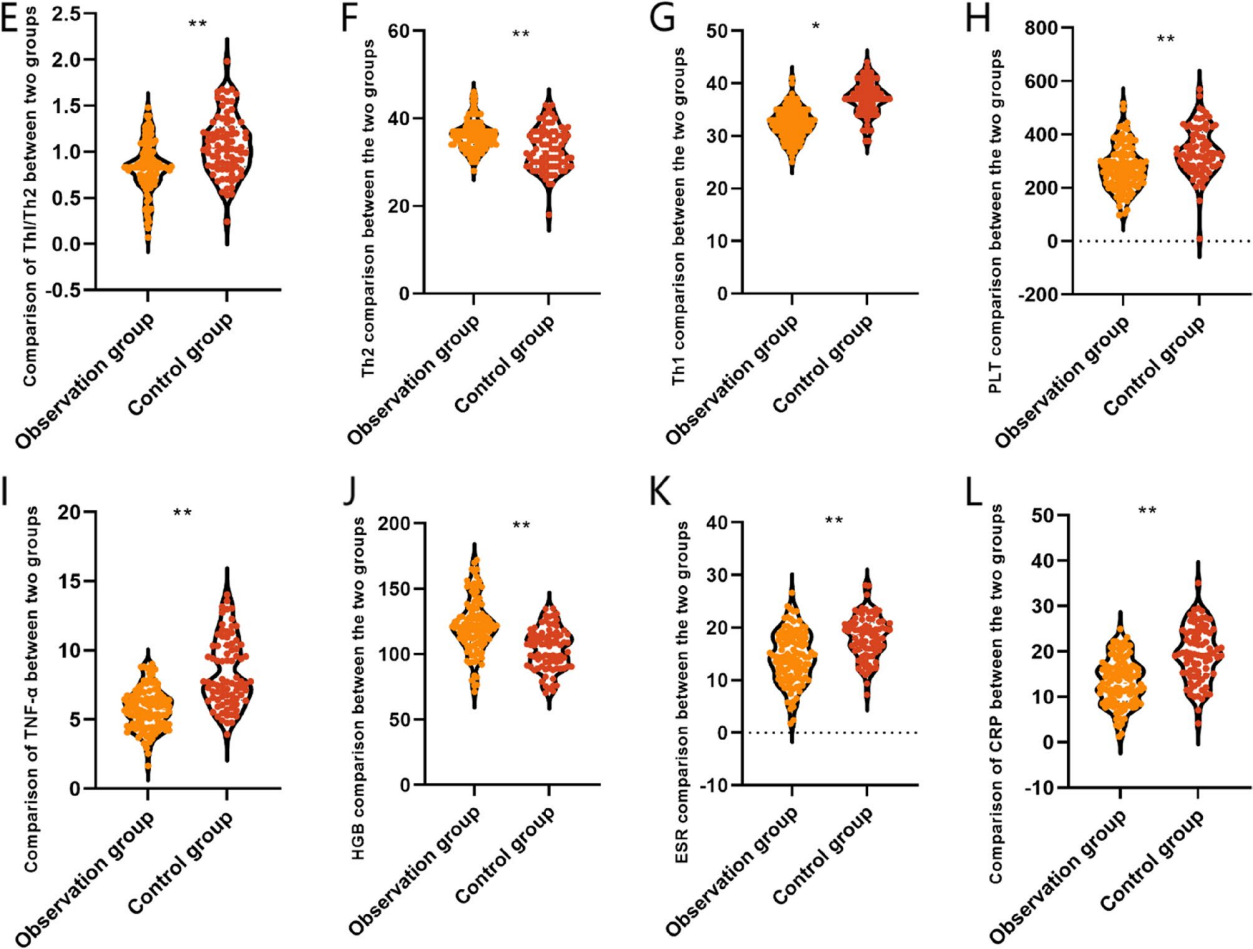
Comparison of serum indexes in two groups of CD patients after treatment Note; TNF-α is tumor necrosis factor-α, CRP is C-reactive protein, ESR is erythrocyte sedimentation rate, HGB is hemoglobin concentration, and PLT is platelet; Data are presented as mean ± SD. *P < 0.05, **P < 0.001

After treatment, TNF-α (5.73 ± 1.45 vs. 8.36 ± 2.47 ng/L), CRP (13.31 ± 5.15 vs. 19.41 ± 6.04 mg/L), ESR (14.34 ± 4.91 vs. 18.21 ± 4.07 mm/h), HGB (121.70 ± 21.52 vs. 103.86 ± 17.28 g/L), PLT (274.71 ± 82.09 vs. 337.52 ± 90.76 × 10⁹/L), Th1 (32.34 ± 2.90 vs. 37.15 ± 3.17%), Th2 (36.10 ± 3.43 vs. 32.94 ± 4.80%) and Th1/Th2 (0.83 ± 0.27 vs. 1.07 ± 0.32) were compared, the difference was statistically significant (*P* < 0.001), demonstrating that the combination of IFX and mesalazine improved the serum indexes of the patients to a higher extent, see Fig. 2.

### Comparison of major symptoms and stool condition between two groups of CD patients

The main symptoms and stool condition are important indicators for assessing the improvement of clinical symptoms in CD patients. The disappearance rate of abdominal pain (73.91% vs. 48.78%), disappearance rate of diarrhea (80.87% vs. 56.10%) and normal rate of stool (77.39% vs. 54.88%) in the observation group were higher than that of the control group, but the number of stools (1.85 ± 0.40 times/d vs. 2.67 ± 1.27 times/d) was lower than that of the control group, and the difference was statistically significant (*P* < 0.001), demonstrating that the combination of IFX and mesalazine can more effectively improve the clinical symptoms of patients, see Table 4.

**Table 4:** Comparison of main symptoms and stool status of two groups of CD patients

Index	Observation group (*n* = 115)	Control group (*n* = 82)	χ²/t value	*P* value
Abdominal pain disappears (n, %)	85 (73.91)	40 (48.78)	13.038	< 0.001
Disappearance of diarrhea (n, %)	93 (80.87)	46 (56.10)	14.140	< 0.001
Number of stools (times/d)	1.85 ± 0.40	2.67 ± 1.27	6.493	< 0.001
Normal stool (n, %)	89 (77.39)	45 (54.88)	11.153	0.001

### Comparison of clinical efficacy between two groups of CD patients

Clinical efficacy is used to assess the efficacy of drugs, the higher the total effective rate, suggesting that the drug treatment effect is more ideal. The total clinical efficacy rate of the observation group (92.17% vs. 80.49%) was higher than that of the control group, and the difference was statistically significant (*P* < 0.05), which indicated that the combination of IFX and mesalazine had a higher clinical efficacy, as shown in Table 5.

**Table 5:** Comparison of clinical efficacy of two groups of CD patients (n, %)

Index	Observation group (*n* = 115)	Control group (*n* = 82)	χ² value	*P* value
Clinical remission	60 (52.17)	36 (43.90)	-	-
Clinical response	46 (40.00)	30 (36.58)	-	-
No response/relapse	9 (7.83)	16 (19.51)	-	-
Total effective rate	106 (92.17)	66 (80.49)	5.900	0.015

### Incidence of adverse reactions in two groups of CD patients

Adverse reactions are used to assess the safety of drug treatment, and the lower the incidence rate, the higher the safety. The total incidence of adverse reactions in the observation group (8.70% vs. 7.32%) was higher than that in the control group, but the difference was not statistically significant (*P* > 0.05), demonstrating that IFX combined with mesalazine does not significantly increase the incidence of adverse reactions and has a high degree of safety, as shown in Table 6.

**Table 6:** Adverse reactions in two groups of CD patients (n, %)

Index	Observation group (*n* = 115)	Control group (*n* = 82)	χ² value	*P* value
Rash	3 (2.61)	2 (2.44)	-	-
Dizziness	4 (3.48)	1 (1.22)	-	-
Nausea	2 (1.74)	1 (1.22)	-	-
Vomit	1 (0.87)	2 (2.44)	-	-
Total incidence	10 (8.70)	6 (7.32)	0.122	0.727

## Discussion

CD pathogenesis has not been fully elucidated, the lesion site can involve any part of the digestive tract including the anus, and the lesion range often presents jumping distribution characteristics, at present, the role of some inhibitors, biologics, hormones and other drugs in symptomatic treatment has been confirmed one after another, and mesalazine is often applied in clinic, but the efficacy of single-use therapy has not reached the ideal state [19, 20]. Therefore, the adoption of safe and effective combination drug therapy is significant for CD patients. Clinical studies have indicated that IFX may reduce immunogenicity, while the local anti-inflammatory effect of mesalazine may reduce the production of drug antibodies, and the combination therapy may act synergistically through different pathways. And through the data of this study, the improvement of intestinal flora, inflammatory indexes, coagulation function, immune response, and clinical symptoms of the patients in the observation group after treatment was better than that of the control group, and the total clinical effective rate of the observation group was higher than that of the control group, and the difference was statistically significant (*P* < 0.05), which indicated that the efficacy of the treatment of IFX in combination with mesalazine was higher than that of the monotherapy of mesalazine. Xu S et al. [21] said that bifidobacteria and Lactobacillus play a protective role through anti-inflammation, maintenance of barrier function, and regulation of immunity; Enterococcus and Enterobacteriaceae may exacerbate inflammation through activation of pro-inflammatory pathways (e.g., NF-κB), and imbalance of bacterial flora may lead to increased intestinal permeability (“leaky gut”), and activation of aberrant immune responses by bacterial antigenic translocation. Previous studies have indicated that inflammatory markers such as TNF-α, CRP, ESR, etc., are associated with intestinal mucosal injury and systemic inflammation, reflecting disease activity; coagulation-related indexes such as HGB, PLT, etc., are all closely associated with chronic inflammatory response of the organism; Th1, Th2, Th1/Th2 reflect the immune response, and there is a mechanism of correlation between flora, immunity, and inflammation, in which dysbiosis of the intestinal flora → activation of intrinsic immunity → release of inflammatory factors → promotion of immune response → persistent inflammation of the intestinal mucosa → clinical symptoms (abdominal pain, diarrhea) and serum index abnormalities, which together affect the occurrence and development of CD, and therefore, using it as a therapeutic target can improve the clinical outcome of patients [22–24].

Ter Avest MM et al. [25] who stated that mesalazine, also known as 5-aminosalicylic acid, is an organic compound that has a significant inhibitory effect on intestinal wall inflammation, inhibiting inflammation-causing prostaglandin synthesis and the formation of inflammatory mediators leukotrienes, and decreasing the destruction of commensal bacteria by pro-inflammatory factors and the over-proliferation of pathogenic bacteria. At the same time, its metabolites may directly promote beneficial bacterial colonization. We hypothesize that mesalazine may block the NF-κB signaling pathway, inhibit cyclooxygenase, reduce the release of inflammatory mediators such as prostaglandins and leukotrienes, and thus potentially inhibit the over-activation of Th1 cells, promote the anti-inflammatory response mediated by Th2 cells, and restore the Th1/Th2 balance. And reference to related studies indicated that mesalazine improves symptoms such as diarrhea and abdominal pain, promotes the recovery of intestinal absorption function, and improves bowel movement by repairing the intestinal mucosal barrier and reducing the stimulation of inflammatory mediators on intestinal smooth muscle and nerves [26]. IFX is the first anti-TNF-α biologic approved and marketed by the U.S. Food and Drug Administration, and TNF-α is an important inflammatory factor, which is mainly produced by activated mononuclear macrophages and T cells, which can promote neutrophil phagocytosis. Higher concentrations of TNF-α were found in diseased mucosal tissues, blood, and feces of CD patients, and this factor disrupts the intestinal barrier function, leading to bacterial translocation, which is associated with intestinal mucosal lesions [27, 28]. In contrast, IFX inhibits the downstream inflammatory signaling pathway and prevents its downstream pro-inflammatory cytokine release by specifically binding to TNF-α and blocking its binding to the receptor, which in turn exerts an anti-inflammatory effect, with a decrease in inflammatory cell infiltration, a subsequent decrease in ESR, and a restoration of erythropoietin activity. Previous studies indicated that IFX induces the programmed death of T cells and monocytes, which play an important role in the pathogenesis of CD, by activating antibody-dependent cell-mediated cytotoxicity and complement-dependent cytolysis effects, thus reducing apoptosis of intestinal tissue cells, promoting Th2 cytokine secretion, balancing the Th1/Th2 ratio, and attenuating the over-immune response [29]. IFX significantly reduces adhesion molecules and reduces chemokine bioactivity, inhibits inflammatory cell chemotactic movement, reduces TNF-α permeability damage to intestinal epithelium, repairs damaged mucosa, improves the intestinal microenvironment, promotes the proliferation of bifidobacteria, Lactobacillus and other beneficial bacteria, inhibits the growth of enterococci, Enterobacteriaceae, and other conditional pathogens, and has a certain protective effect on the intestinal mucosal barrier and improves clinical symptoms.

We propose that IFX and mesalazine may act on different parts of the inflammatory cascade (upstream mediators and core cytokines), forming a complementary, comprehensive inhibition of the inflammatory network; combining the mucosal repair effect of mesalazine with the systemic anti-inflammatory effect of IFX to alleviate the deep tissue lesions, reduce permeability, promote epithelial repair, and reduce bacterial translocation. We speculate that mesalazine may be able to reduce local inflammation, reduce the recognition of IFX by the immune system, prolong the effective blood concentration of IFX, enhance the efficacy and delay drug resistance, and synergize the antioxidant effect of mesalazine with the antifibrotic effect of IFX, both of which are synergistic in multi-dimensional ways through complementary anti-inflammatory targets, combination of local and systemic effects, immunomodulation, and reduction of drug resistance, to achieve a more comprehensive control of the disease. These mechanistic hypotheses require prospective experimental validation. Previous data have shown that drug combinations may cause an increase in clinical adverse reactions and a decrease in safety in terms of pharmacokinetics, pharmacodynamics, drug-drug interactions and individual patient differences [30, 31]. However, the data of this study showed that the adverse reactions such as rash, dizziness, nausea, and vomiting in the observation group were higher than those in the control group, but the difference was not statistically significant (*P* > 0.05), which suggests that the combination of IFX and mesalazine does not significantly increase the risk of adverse reactions, and it may benefit from the complementary mechanism of drug action, drug interactions, and provide a safer and more effective therapeutic option for patients with CD. However, the clinical application of IFX combined with mesalazine in CD patients has rarely been reported, and further studies are needed to verify the scientific validity of this study.

Notably, the culture-based results were consistent with 16 S rRNA sequencing data, which showed increased beneficial bacteria and decreased conditionally pathogenic bacteria, validating the reliability of our culture-based findings. Recent evidence from clinical trials [34, 35] supports the potential benefits of combination therapy approaches in inflammatory bowel disease management.

Despite the above research value, there are still limitations: As a retrospective study, there is an inherent risk of selection bias and confounding despite our efforts to control for major covariates through multivariate regression and IPTW. Unmeasured confounders may still exist. The small sample size of the study may lead to increased statistical errors, and it is difficult to verify the significance of the differences between groups, for example, there may be a bias in judging the efficacy of the drug or the risk of complications, and it is necessary to optimize the level of evidence by expanding the size of the data through multi-center cooperation or extending the study period. This study provides 40-week medium-term observations; we have planned to extend follow-up to 104 weeks to provide additional data on mucosal healing and relapse rates. The disease duration of CD can be decades, so short-term follow-up is unable to assess the impact of recurrent disease activity on long-term outcomes, such as intestinal fibrosis and cancer.

## Conclusion

In conclusion, infliximab combined with mesalazine demonstrates superior clinical efficacy compared to mesalazine monotherapy in patients with Crohn’s disease. This combination effectively improves intestinal microbiota balance, reduces inflammation, modulates immune responses, and alleviates clinical symptoms without significantly increasing adverse events. These findings support the safety and therapeutic potential of IFX-mesalazine co-treatment as a promising strategy in the management of Crohn’s disease. However, further large-scale and long-term studies are warranted to validate these outcomes. [15]

## Supplementary Information

Supplementary Material 1: Figure S1. Comprehensive analysis of gut microbiota composition and diversity in Crohn's disease patients treated with IFX + mesalazineversus mesalazine alone.Alpha diversity indices comparing pre-treatmentand post-treatmentsamples.Shannon diversity index showing species richness and evenness.Simpson diversity index indicating community diversity.Chao1 index representing species richness. Box plots display median, interquartile range, 1.5× interquartile range, and outliers.Beta diversity analysis using Principal Coordinates Analysisbased on Bray-Curtis dissimilarity matrix. Each point ACCEPTED MANUSCRIPT Accepted manuscript represents an individual sample; ellipses indicate 95% confidence intervals. Percentage values on axes represent the proportion of variance explained by each principal coordinate.Relative abundance of bacterial phyla in post-treatment samples. Bar width represents 100% of the bacterial community. Colors indicate different phyla: Actinobacteria, Bacteroidetes, Firmicutes, Others, and Proteobacteria. Pre-treatment and post-treatment comparisons are shown for each group.Heatmap displaying relative abundance of the top 10 bacterial genera. Color intensity represents abundance percentage, with red indicating higher abundance and purple indicating lower abundance. Genera are listed in italics on the y-axis.Differential abundance analysis showing log2 fold change of significantly different genera between groups. Orange bars indicate genera enriched in the Observation group; blue bars indicate genera enriched in the Control group. Only genera with significant differencesafter false discovery rate correction are shown. Statistical significance: ***p < 0.001

Supplementary Material 2.

## Data Availability

The datasets used and/or analyzed during the current study are available from the corresponding author on reasonable request.
