# Supplementary figures and images for: Therapeutic regimen of Crohn’s disease: effect of Infliximab combined with mesalazine on intestinal flora and inflammatory indexes in patients
Source: BMC Gastroenterol. 2025 Sep 29;25:687. doi: 10.1186/s12876-025-04236-9 (PMC12482166; doi:10.1186/s12876-025-04236-9)

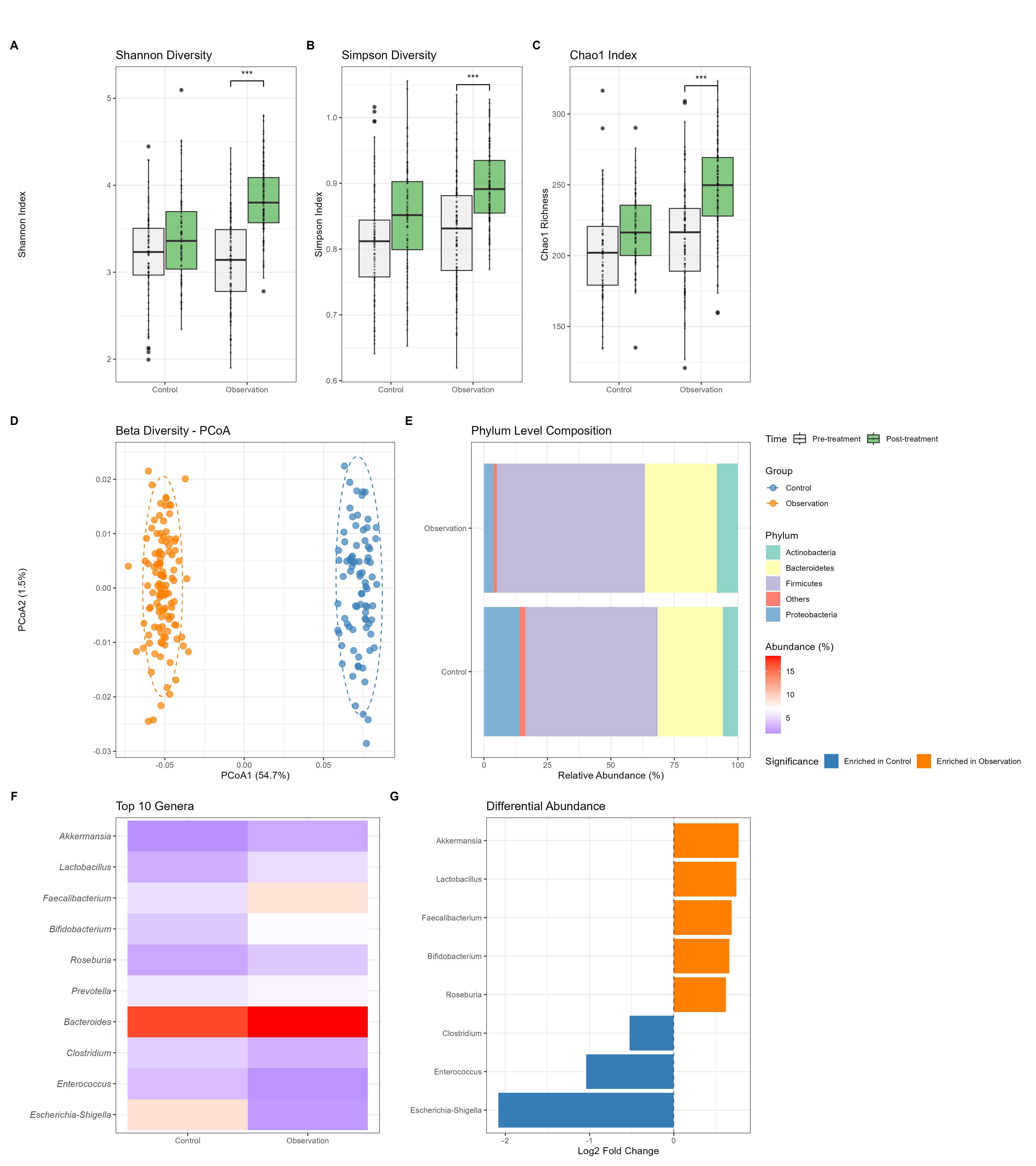

Supplement: Supplementary file 1 — Supplementary Material 1: Figure S1. Comprehensive analysis of gut microbiota composition and diversity in Crohn's disease patients treated with IFX + mesalazineversus mesalazine alone.Alpha diversity indices comparing pre-treatmentand post-treatmentsamples.Shannon diversity index showing species richness and evenness.Simpson diversity index indicating community diversity.Chao1 index representing species richness. Box plots display median, interquartile range, 1.5× interquartile range, and outliers.Beta diversity analysis using Principal Coordinates Analysisbased on Bray-Curtis dissimilarity matrix. Each point ACCEPTED MANUSCRIPT Accepted manuscript represents an individual sample; ellipses indicate 95% confidence intervals. Percentage values on axes represent the proportion of variance explained by each principal coordinate.Relative abundance of bacterial phyla in post-treatment samples. Bar width represents 100% of the bacterial community. Colors indicate different phyla: Actinobacteria, Bacteroidetes, Firmicutes, Others, and Proteobacteria. Pre-treatment and post-treatment comparisons are shown for each group.Heatmap displaying relative abundance of the top 10 bacterial genera. Color intensity represents abundance percentage, with red indicating higher abundance and purple indicating lower abundance. Genera are listed in italics on the y-axis.Differential abundance analysis showing log2 fold change of significantly different genera between groups. Orange bars indicate genera enriched in the Observation group; blue bars indicate genera enriched in the Control group. Only genera with significant differencesafter false discovery rate correction are shown. Statistical significance: ***p < 0.001 [file 12876_2025_4236_MOESM1_ESM.png]
