# Supplementary material for: Therapeutic regimen of Crohn’s disease: effect of Infliximab combined with mesalazine on intestinal flora and inflammatory indexes in patients
Source: BMC Gastroenterol. 2025 Sep 29;25:687. doi: 10.1186/s12876-025-04236-9 (PMC12482166; doi:10.1186/s12876-025-04236-9)
Supplement: Supplementary file 2 — Supplementary Material 2. [file 12876_2025_4236_MOESM2_ESM.docx]

**Table S1 16S rRNA gene sequencing results: bacterial diversity and composition**

| **Parameter** | **Observation group (n=115)** | **Control group (n=82)** | **P value** |
| --- | --- | --- | --- |
| **Alpha Diversity (Post-treatment)** |  |  |  |
| Shannon index | 3.82±0.45 | 3.41±0.52 | <0.001 |
| Simpson index | 0.89±0.06 | 0.85±0.08 | <0.001 |
| Chao1 richness | 245.7±32.4 | 218.3±29.6 | <0.001 |
| **Phylum Level (%)** |  |  |  |
| Firmicutes | 58.3±7.2 | 52.1±8.4 | <0.001 |
| Bacteroidetes | 28.4±5.6 | 25.7±6.1 | 0.002 |
| Actinobacteria | 8.2±2.3 | 5.9±2.1 | <0.001 |
| Proteobacteria | 3.8±1.4 | 14.2±3.7 | <0.001 |
| Others | 1.3±0.8 | 2.1±1.2 | 0.089 |
| **Genus Level (Top 10, %)** |  |  |  |
| Bifidobacterium | 6.8±1.9 | 4.3±1.7 | <0.001 |
| Lactobacillus | 5.2±1.6 | 3.1±1.4 | <0.001 |
| Bacteroides | 18.3±4.2 | 16.7±3.8 | 0.007 |
| Faecalibacterium | 8.7±2.1 | 5.4±1.8 | <0.001 |
| Roseburia | 4.3±1.2 | 2.8±0.9 | <0.001 |
| Escherichia-Shigella | 2.1±0.8 | 8.9±2.3 | <0.001 |
| Enterococcus | 1.8±0.6 | 3.7±1.1 | <0.001 |
| Clostridium | 3.2±0.9 | 4.6±1.3 | <0.001 |
| Prevotella | 6.4±2.3 | 5.8±2.1 | 0.064 |
| Akkermansia | 2.9±1.1 | 1.7±0.8 | <0.001 |
| **Functional Predictions (KEGG Level 2)** |  |  |  |
| Carbohydrate metabolism | 12.4±1.8 | 10.9±1.6 | <0.001 |
| Amino acid metabolism | 10.2±1.4 | 9.8±1.3 | 0.042 |
| Lipid metabolism | 4.8±0.9 | 6.2±1.1 | <0.001 |
| Energy metabolism | 5.6±0.8 | 4.9±0.7 | <0.001 |
| Biosynthesis of secondary metabolites | 8.3±1.2 | 7.1±1.0 | <0.001 |

Note: Data are presented as mean ± SD. Alpha diversity indices were calculated at the OTU level with 97% similarity. Relative abundances at phylum and genus levels are shown as percentages of total reads. Functional predictions were performed using PICRUSt2. KEGG = Kyoto Encyclopedia of Genes and Genomes.

**Table S2 Baseline characteristics and main outcomes after inverse probability of treatment weighting (IPTW)**

| **Characteristics** | **Before IPTW** | **After IPTW** |
| --- | --- | --- |
|  | Observation group (n=115) | Control group (n=82) |
| Age (years) | 31.93±5.96 | 31.56±5.57 |
| Male sex (%) | 62.61 | 60.98 |
| BMI (kg/m²) | 23.75±1.48 | 23.39±1.56 |
| Disease duration (years) | 7.20±2.98 | 7.12±2.55 |
| Baseline CDAI | 285.4±52.3 | 278.9±48.7 |
| Previous biologics (%) | 15.7 | 12.2 |
| **Main Outcomes** |  |  |
| Clinical remission (%) | 52.17 | 43.90 |
| Total effective rate (%) | 92.17 | 80.49 |
| TNF-α reduction (ng/L) | 17.43±3.87 | 14.45±3.92 |
| CRP reduction (mg/L) | 24.90±6.21 | 18.48±5.86 |

Note: SMD = standardized mean difference; IPTW = inverse probability of treatment weighting; BMI = body mass index; CDAI = Crohn's Disease Activity Index; TNF-α = tumor necrosis factor-α; CRP = C-reactive protein. An SMD <0.1 indicates good balance between groups.
